# Long Noncoding RNA HAGLROS Promotes the Malignant Progression of Bladder Cancer by Regulating the miR-330-5p/SPRR1B Axis

**DOI:** 10.3389/fonc.2022.876090

**Published:** 2022-05-18

**Authors:** Shiwei Xiao, Yigang Zuo, Yanan Li, Yinglong Huang, Shi Fu, Dongbo Yuan, Xuhua Qiao, Haifeng Wang, Jiansong Wang

**Affiliations:** ^1^ Department of Urology, The Second Affiliated Hospital of Kunming Medical University, Yunnan Institute of Urology, Kunming, China; ^2^ Department of Urology, Guizhou Provincial People’s Hospital, Guiyang, China; ^3^ Department of Basic Chemistry, College of Pharmacy, Guizhou University of Traditional Chinese Medicine, Guiyang, China

**Keywords:** bladder cancer, HAGLROS, miR-330-5p, SPRR1B, oncogene

## Abstract

Bladder cancer (BC) is the most common genitourinary malignancy worldwide, and its aetiology and pathogenesis remain unclear. Accumulating evidence has shown that HAGLROS is closely related to the occurrence and progression of various cancers. However, the biological functions and underlying mechanisms of HAGLROS in BC remain unknown. In the present study, the expression of HAGLROS in BC was determined by public dataset analysis, transcriptome sequencing analysis, qRT–PCR and ISH assays. Gain- or loss-of-function assays were performed to study the biological roles of HAGLROS in BC cells and nude mouse xenograft model. Bioinformatic analysis, qRT–PCR, western blot, immunohistochemistry, FISH assays, subcellular fractionation assays and luciferase reporter assays were performed to explore the underlying molecular mechanisms of HAGLROS in BC. Here, we found that HAGLROS expression is significantly upregulated in BC tissues and cells, and elevated HAGLROS expression was related to higher pathologic grade and advanced clinical stage, which is significant for BC diagnosis. HAGLROS can enhance the growth and metastasis of BC *in vitro* and *in vivo*. Furthermore, miR-330-5p downregulation reversed the BC cells proliferation, migration and invasion inhibited by silencing HAGLROS. SPRR1B silencing restored the malignant phenotypes of BC cells promoted by miR-330--5p inhibitor. Mechanistically, we found that HAGLROS functions as a microRNA sponge to positively regulate SPRR1B expression by sponging miR-330-5p. Together, these results demonstrate that HAGLROS plays an oncogenic role and may serve as a potential biomarker for the diagnosis and treatment of BC.

## Introduction

BC is the most common genitourinary malignancy worldwide. In the past decade, the morbidity and mortality of BC have increased significantly, showing a gradually decreasing trend in age (1). Non-muscle-invasive bladder cancer (NMIBC) can be treated with transurethral resection of the bladder tumour and postoperative adjuvant chemotherapy/immunotherapy. Of note, approximately 50-70% of patients with NMIBC will relapse, and 10-20% will eventually progress to muscle-invasive bladder cancer (MIBC) or metastasize (2). Although MIBC is mainly treated with radical cystectomy, there are many short-term and long-term complications that cause considerable pain to patients (3, 4). However, the aetiology and pathogenesis of BC remain unclear. Therefore, an in-depth understanding of the underlying mechanisms and identifying effective early detection biomarkers have great clinical significance for improving the diagnosis and therapeutic strategies of BC.

LncRNAs are noncoding RNAs greater than 200 nucleotides in length that were initially ignored as “transcription noises” due to a lack of protein-coding function (5). However, the rapid development of RNA genomics technology highlights the notion that lncRNAs are key players in gene expression regulation and significantly contribute to human disease progression, especially in cancers (6). In the past decade, growing evidence has shown that lncRNAs play a vital role in gene expression and diverse biological processes by regulating transcription, sponging miRNAs, and modifying epigenetic regulation (6, 7). Notably, enormous amounts of lncRNAs have been found to play an important role in invasion, metastasis and drug resistance in BC (8–10). For instance, lncRNA-SNHG1 promotes basal MIBC cell invasion by interacting with the PP2A catalytic subunit and inducing autophagy (11). LncRNA-SLC16A1-AS1 induces metabolic reprogramming as a target and coactivator of E2F1 in BC progression (12). HAGLR opposite strand lncRNA (HAGLROS), which is located on chromosome 2q31.1, is a 699-bp lncRNA that was first reported in 2018 (13). This study revealed that HAGLROS can promote tumorigenesis and progression *via* mTOR signal-mediated inhibition of autophagy in gastric cancer. Moreover, recent study showed that HAGLROS can promote proliferation and angiogenesis and inhibit apoptosis by activating the Erk1/2 and AKT or JNK signalling pathways in LSCC cells (14). It was reported that HAGLROS could promote osteosarcoma invasion and metastasis through the ROCK1 signalling pathway (15). Subsequently, accumulating evidence has shown that HAGLROS is closely associated with proliferation, invasion, autophagy and drug resistance, and plays a vital role in the occurrence and progression of various cancers. However, the biological functions and underlying mechanisms of HAGLROS in BC remain unknown.

In the present study, the expression level, biological function and underlying mechanism of HAGLROS in BC were initially investigated. Our findings reveal that HAGLROS was significantly upregulated in BC tissues and cells compared with adjacent normal tissues and cells, which was verified by online databases and our transcriptome sequencing dataset, qRT–PCR and *in situ* hybridization (ISH) assay. Furthermore, HAGLROS can enhance the growth and metastasis of BC *in vitro* and *in vivo*. Knockdown of miR-330-5p promotes small proline rich protein 1B (SPRR1B) expression and reverses the malignant phenotypes inhibition of BC induced by silencing HAGLROS. SPRR1B silencing restored the malignant phenotypes of BC cells promoted by decreased miR-330-3p. Mechanistically, we found that HAGLROS was mostly distributed in the cytoplasm and positively regulated SPRR1B expression by sponging miR-330-5p in a competing endogenous RNA (ceRNA)-dependent manner. Overall, our results suggest that the HAGLROS/miR-330-5p/SPRR1B axis is a promising novel biomarker that may serve as a powerful therapeutic and diagnostic target in BC.

## Materials and Methods

### Clinical Samples

From June 2019 to September 2020, 43 pairs of fresh BC tissues and corresponding non-tumour tissues were collected from patients who underwent radical cystectomy. The samples were immediately cleaned with normal saline and snap-frozen in liquid nitrogen after resection. All samples were confirmed independently by two pathologists. The patients were informed of the study contents and signed informed consent forms. This study was approved and supported by the Ethics Committee of the Second Affiliated Hospital of Kunming Medical University.

### Cell Culture

The BC cell lines T24 and 5637 and the normal bladder uroepithelium cell line SV-HUC-1 and human embryonic kidney (HEK) 293T were purchased from Procell Life Science & Technology Co., Ltd. (Wuhan, China). T24, SV-HUC-1 and HEK-293T cells were cultured in DMEM (Gibco, NY, USA) with 10% foetal bovine serum (Gibco, NY, USA), 5637 cells were cultured with RPMI-1640 medium (Gibco, NY, USA) and 10% foetal bovine serum (FBS). All cells were cultured at 37°C in a humidified 5% CO_2_ atmosphere.

### RNA Extraction and Quantitative Real-Time PCR (qRT–PCR)

Total RNA was extracted from bladder tissue and cells using TRIzol reagent (Thermo Fisher Scientific, MA, USA) according to the manufacturer’s instructions. The sequence of lncRNA/mRNA primers listed in **Supplementary Table 1**. The miRNA primers were designed and synthesized by RiboBio Biotechnology (Guangzhou, China). QRT–PCR assays were performed using qRT–PCR Starter Kits (RiboBio, Guangzhou, China) and run in a LightCycler 96 sequence detection instrument (Roche, Basel, Switzerland) according to the manufacturer’s instructions. Expression was normalized to GAPDH, U6 and β-actin. Each experiment was repeated at least thrice.

### *In Situ* Hybridization (ISH)

ISH assay was used to detect the HAGLORS expression in the bladder tissue microarray. The HAGLROS probes were designed and synthesized by Boster Biological Technology (Wuhan, China). The images were analysed by ImageScope (Leica, Mannheim, Germany) software and the expression level of HAGLORS was evaluated by histochemistry score (H-Score).

### RNA Fluorescent *In Situ* Hybridization (FISH) and Subcellular Fractionation Assay

FISH was performed to detect the subcellular colocalization of HAGLROS, miR-330-5p and SPRR1B. A FISH kit was purchased from Focofish Biotechnology (Guangzhou, China). The U6 probes were purchased from Sangon (Shanghai, China). Cell nuclei were counterstained with DAPI (Sangon, Shanghai, China). The cytoplasmic and nuclear fractions of T24 and 5637 cells were extracted using nuclear and cytoplasmic extraction reagents (Thermo Fisher Scientific, MA, USA) according to the manufacturer’s protocol. Then, the RNA was extracted, and qRT–PCR was performed as previously described. Each experiment was repeated at least three times.

### Transcriptome Sequencing

Briefly, total RNA was extracted from three pairs of fresh bladder cancer tissues and corresponding adjacent tissues. The eligible RNAs were sent to the sequencing company for quality control, library construction and sequencing.

### Cell Transfection

The plasmids of overexpressing HAGLROS (OE-HAGLROS), short hairpin (sh) HAGLROS and sh-SPRR1B were designed and synthesized by GenoMeditech Biotechnology (Shanghai, China). T24 and 5637 cells were transfected. The cells were harvested 48-72 hours. Puromycin (Solarbio, Beijing, China) was used to screen stably transfected cells. The transfection efficiency was evaluated by qRT–PCR. The miR-330-5p mimics/inhibitors were purchased from RiboBio Biotechnology (Guangzhou, China). Each experiment was repeated at least three times.

### Western Blot Assay

Western blot assay was used to detect protein expression. Tissues and cells were lysed with ice-cold RIPA lysis buffer (Beyotime, Shanghai, China). The protein was quantified using a Bradford Protein Assay Kit (Beyotime, Shanghai, China), separated by sodium dodecyl sulfate–polyacrylamide gel electrophoresis (Beyotime, Shanghai, China), transferred to a polyvinylidene fluoride (PVDF) membrane (Millipore, MA, USA), blocked with bovine serum albumin (Solarbio, Beijing, China), hybridized with SPRR1B (1:1000, Abcam, MA, USA) and GAPDH (1:1000, Abmart, Shanghai, China), and incubated with a secondary antibody (1:2000, Cell Signaling Technology, MA, USA). Signals were visualized with an enhanced chemiluminescence (ECL) kit (Beyotime, Shanghai, China). Then, the bands were detected using a gel imager system and analysed by ImageJ software (NIH, Bethesda, MD, USA). Each experiment was repeated at three times.

### Cell Counting Kit-8 (CCK-8) Assay

BC cell proliferation was observed using an Enhanced Cell Counting Kit-8 (Beyotime, Shanghai, China) according to the manufacturer’s instructions. Briefly, 100 µl cell suspension was seeded onto 96-well plates per well. After 0 h, 24 h, 48 h and 72 h, 10 µl CCK-8 solution was added to each well, and the cells were incubated for 1 hour. Finally, the absorbance was measured at 450 nm using a microplate reader (Thermo Fisher Scientific, MA, USA). Each experiment was repeated at least three times.

### Wound Healing Assay

The migration capability of cells was tested by wound healing assay. The wounds were scratched using a 200-μl pipette tip in approximately 90% confluent cells. The migration of cells was acquired using a microscope (Olympus, Tokyo, Japan). Finally, wound healing areas were measured by ImageJ software (NIH, Bethesda, MD, USA). Each experiment was repeated at least three times.

### Transwell Assay

The invasive capability of cells was observed using Transwell assays. Briefly, 100 µl diluted Matrigel (Corning, MA, USA) was added to the upper chamber of the Transwell (Corning, MA, USA), and the transwells were incubated at 37°C for 1 hour. Then, 200 μl serum-free medium with 5×10^4^ cells were seeded into each upper chamber. Culture medium containing 10% FBS was added to the lower chamber. The cells in the upper chamber were wiped using cotton after 48 hours of incubation, fixed with paraformaldehyde, and stained with crystal violet. The number of invaded cells was recorded under a microscope. Each experiment was repeated at least three times.

### Flow Cytometry Assay

The cell cycle was determined by using a Cell Cycle Analysis Kit (Beyotime, Shanghai, China) according to the manufacturer’s instructions. The cell cycle was assessed by flow cytometry. Each experiment was repeated at least three times.

### Immunohistochemistry (IHC)

Protein expression levels were detected by immunohistochemical staining. The bladder tissues were prepared into paraffin sections. Then, these sections were deparaffinized, used for antigen retrieval, and incubated with a primary antibody against SPRR1B (1:150, Proteintech, Wuhan, China) overnight at 4°C. A universal two-step assay kit (Zsboi, Beijing, China) was used for IHC. The immunostaining images were detected using an optical microscope. Each experiment was repeated at least three times.

### Dual-Luciferase Reporter Assays

Luciferase reporter plasmids were designed and synthesized by GenoMeditech (Shanghai, China). HEK-293T cells were cotransfected with luciferase reporter plasmids and microRNAs. A dual-luciferase reporter assay system (Promega, WI, USA) was used to determine the luciferase activity 48 h later according to the instructions. Each experiment was repeated at least three times.

### Tumour Xenograft Implantation in Nude Mice

T24 cells, OE-HAGLROS and sh-HAGLROS stably transfected T24 cells (2 × 10^6^ cells) were subcutaneously inoculated into BALB/c nude mice (18-22 g). Then, tumour volumes were measured every 3 days. The animal experiments were approved and supported by the Ethics Committee of the Second Affiliated Hospital of Kunming Medical University. Each experiment was repeated at least three times.

### Immunofluorescence

Immunofluorescence was used to detect ki67 expression levels in nude mice BC tissues, which was performed according to the standard protocol previously reported. The primary antibody against ki67 was purchased from Proteintech (1:150, Wuhan, China), and the 488-conjugated secondary antibody was purchased from KPL (1:1000, MA, USA). Sections were visualized using a fluorescence microscopy (Olympus, Tokyo, Japan). Each experiment was repeated at least three times.

### Bioinformatics Analysis

The DEGseq2 R package (R version 4.0.1) was used to identify differentially expressed lncRNAs and mRNAs (DE-lncRNAs and DE-mRNAs) in RNA-sequencing data according to the thresholds |log2 (FC) |> 1.0 and *P* value < 0.05. A total of 408 BC samples and 19 normal samples were downloaded from TCGA database (https://portal.gdc.cancer.gov/), and the DE-lncRNAs and DE-mRNAs were identified according to the thresholds |log2 (FC) |> 0.585 and *P* value < 0.05. The DE-lncRNAs and DE-mRNAs were overlapped between the RNA-sequencing data and TCGA data. The differential expression of candidate genes and the relationship of their expression levels were evaluated in data retrieved from the starBase database (https://starbase.sysu.edu.cn/), the GEPIA database (http://gepia.cancer-pku.cn/), the Lnc2Cancer database (http://bio-bigdata.hrbmu.edu.cn/lnc2cancer/) and the LncBase database (https://diana.e-ce.uth.gr/lncbase/).

### Statistical Analyses

The data were obtained from three independent experiments performed at least three time. All results are presented as the mean ± SD. Statistical analysis was performed with one-way ANOVA and Student’s t test using GraphPad Prism 8.0 (GraphPad Software, La Jolla, CA, USA). In this study, *P-values* < 0.05 was considered statistically significant.

## Results

### HAGLROS Expression Is Significantly Upregulated in BC

**Figure 1:**
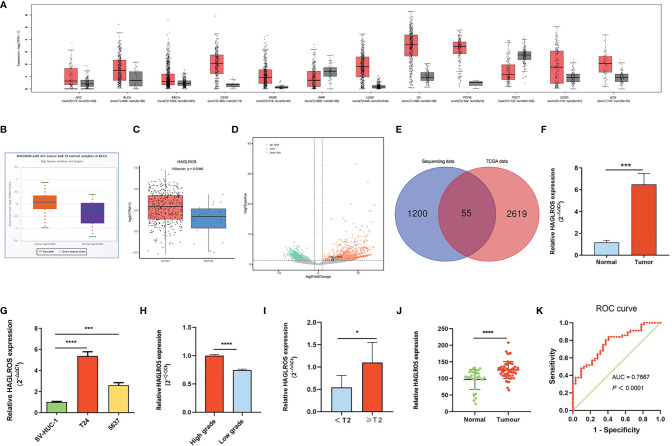
HAGLROS expression is significantly upregulated in BC. **(A)** The expression of HAGLROS in common tumour samples and paired normal tissues was analyzed through the GEPIA database. **(B, C)** The HAGLROS expression level in BC was explored using the starBase database (P<0.0001) and the Lnc2Cancer database (P=0.0046). **(D)** Volcano plot of DE-lncRNAs in our transcriptome sequencing dataset. Each point in the plot represents a gene. Orange dots represent upregulated genes, whereas green dots represent downregulated genes. **(E)** Venn diagram of upregulated DE-lncRNAs based on the overlap between TCGA dataset and our RNA-sequencing dataset. **(F, G)** HAGLROS expression in BC samples and cells was determined using qRT–PCR assays. **(H, I)** HAGLROS expression was determined using qRT–PCR assays in different pathologic grades and clinical stages tissues. **(J)** ISH was performed to detect the expression of HAGLROS in bladder tissue microarray. **(K)** ROC curve for prediction of BC using ISH-based HAGLROS expression level. The AUC was 0.7667, *P*<0.0001. Each experiment was repeated at least three times. *P < 0.05, ***P < 0.001, ****P < 0.0001.

HAGLROS expression in common tumours was analysed using the GEPIA database, and the results showed that HAGLROS was highly expressed in most tumours, including BC, lung squamous cell carcinoma, and cervical squamous cell carcinoma (**Figure 1A**). To further study whether HAGLROS is significantly upregulated in BC, we retrieved and analysed data from the starBase database and the Lnc2Cancer database. The results indicated that HAGLROS was both obviously elevated in BC (**Figures 1B, C**). Meanwhile, our transcriptome sequencing analysis identified 2210 DE-lncRNAs, including 1255 upregulated DE-lncRNAs and 955 downregulated DE-lncRNAs (**Figure 1D**). There were 55 upregulated DE-lncRNAs in both the TCGA dataset and our RNA sequencing (RNA-seq) dataset (**Figure 1E**). These data also confirmed that HAGLROS was significantly overexpressed (log2FoldChange = 5.70, *P*<0.01) in BC tissues (**Supplementary Table 2**). Furthermore, qRT–PCR assays were used to determine the HAGLROS expression levels in BC tissues and cells. We found that HAGLROS was obviously upregulated in BC tissues and cells (**Figures 1F, G** **)**, and increased HAGLROS expression was related to higher pathologic grade and advanced clinical stage (**Figures 1H, I**). To further validate the expression and diagnostic value of HAGLROS, ISH assay was used to detect the HAGLROS expression level of bladder tissue microarray. The results also showed that HAGLROS was significantly overexpressed in BC (**Figure 1J**). Subsequently, the adjacent normal tissues were used as a control to produce a receiver operating characteristic (ROC) curve. The data of ROC curve indicated that HAGLROS was helpful for the diagnosis of BC (**Figure 1K**). In conclusion, these results demonstrate that HAGLROS expression is significantly upregulated in BC, which is valuable for the diagnosis of BC.

### HAGLROS Plays an Oncogenic Role in BC

**Figure 2:**
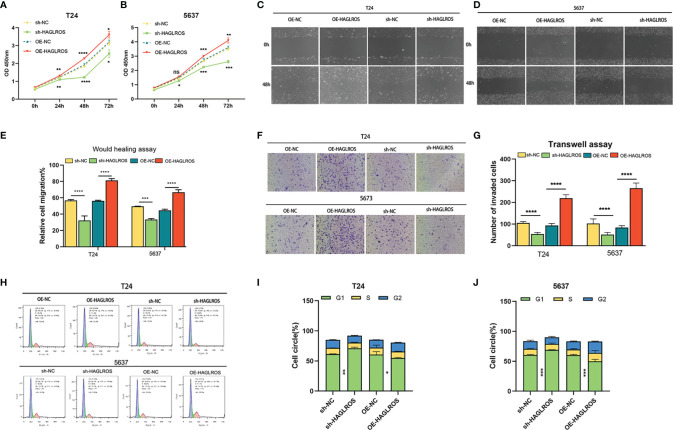
HAGLROS promotes the proliferation, migration and invasion of BC *in vitro*. **(A, B)** Cell viability was assessed by CCK-8 assays in T24 and 5637 BC cells. **(C–E)** Cell migration was determined by wound healing assays in T24 and 5637 BC cells (magnification, x40). **(F, G)** Cell invasion was monitored by Transwell assays in T24 and 5637 BC cells (magnification, x100). **(H–J)** The cell cycle changes of T24 and 5637 BC cells were investigated using flow cytometry assays. *P < 0.05, **P < 0.01, ***P < 0.001, ****P < 0.0001, ns, not significant.

To further study the biological functions of HAGLROS in BC, we constructed and screened HAGLROS overexpression and knockdown vectors, which were transfected into T24 and 5637 BC cells for subsequent experiments. The CCK-8 assay results indicated that HAGLROS overexpression promoted BC cell proliferation, whereas HAGLROS knockdown inhibited BC cell proliferation (**Figures 2A, B**). To investigate the role of HAGLROS in BC, wound healing assays and Transwell assays were performed. The results revealed that upregulated HAGLROS expression strengthened BC cell migration and invasion, while downregulated HAGLROS expression decreased BC cell migration and invasion (**Figures 2C–G**). Moreover, the flow cytometry data showed that knockdown of HAGLROS induced G1-phase arrest in T24 and 5637 cells (**Figures 2H–J**). Collectively, these results reveal that HAGLROS promotes the proliferation, migration and invasion of BC cells and plays an oncogenic role *in vitro*.

### HAGLROS Positively Regulates SPRR1B Expression

**Figure 3:**
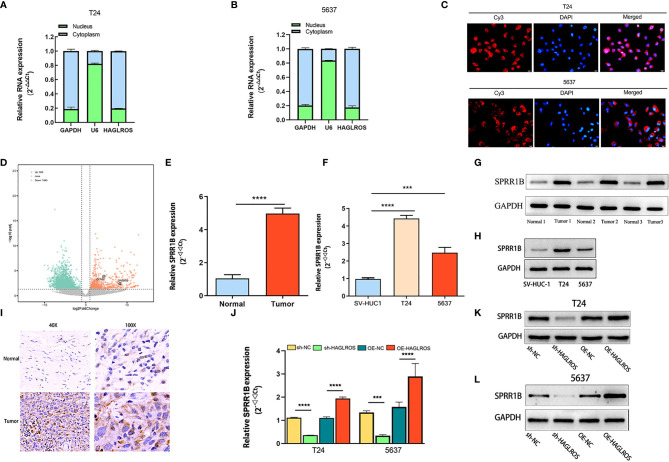
HAGLROS positively regulates SPRR1B expression. **(A, B)** The cytoplasmic and nuclear RNA of T24 and 5637 cells were extracted for qRT–PCR assays. GAPDH was used as a cytoplasmic marker, and U6 was used as a nuclear marker. **(C)** FISH assay was performed to confirm the location of HAGLROS in T24 and 5637 cells (magnification, x 400). HAGLROS (Cy3, red), cell nuclei (DAPI, blue). **(D)** Volcano plot of DE-mRNAs in our transcriptome sequencing dataset. Each point in the plot represents a gene. Orange dots represent upregulated genes, whereas green dots represent downregulated genes. **(E–H)** SPRR1B expression was determined by qRT–PCR assays and western blot assays in bladder tissues and cells. **(I)** SPRR1B expression was analyzed by IHC assays in BC and adjacent normal samples. **(J–L)** The changes of SPRR1B expression were evaluated by qRT–PCR and western blot assays in BC cells transfected with sh-NC, sh-HAGLROS, OE-NC and OE-HAGLROS vectors. Each experiment was repeated at least three times. ***P < 0.001, ****P < 0.0001.

To explore the underlying mechanism of HAGLROS in BC cells, the cytoplasmic and nuclear fractions of T24 and 5637 cells were extracted for qRT–PCR assays, and a FISH assay was performed. Both results showed that HAGLROS was mostly located in the cytoplasm of BC cells (**Figures 3A–C**). Therefore, we hypothesized that HAGLROS functioned as a microRNA sponge in BC. Then, the TCGA dataset and our transcriptional sequencing dataset were used to identify the DE-mRNAs in BC. Our transcriptome sequencing analysis identified 689 differentially upregulated mRNAs in BC (**Figure 3D**). These mRNAs were overlapped, and the top 10 mRNAs in the two datasets were intersected (**Supplementary Figures 1A, B** and **Supplementary Table 3**). The data showed that six mRNAs were highly expressed in both groups, and SPRR1B was the most highly expressed in our sequencing dataset (log2FoldChange=11.83, *P*<0.01). Furthermore, qRT–PCR assay, western blot assay and IHC were used to detect SPRR1B expression levels in BC tissues and cells. The results showed that SPRR1B was highly overexpressed at both the transcriptional and protein levels in BC (**Figures 3E–I**). In addition, increasing HAGLROS expression obviously promoted SPRR1B expression, whereas decreasing HAGLROS expression inhibited SPRR1B expression in BC cells (**Figures 3J–L**). Of note, qRT-PCR results demonstrate that sh-SPRR1B can decrease HAGLROS expression (**Supplementary Figure 2**). These results suggest that HAGLROS positively regulates SPRR1B expression.

### HAGLROS Regulates SPRR1B Expression by Sponging miR-330-5p

**Figure 4:**
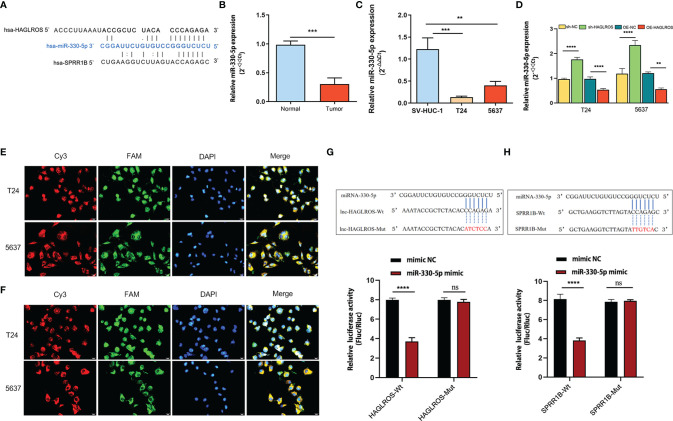
HAGLROS regulates SPRR1B expression by sponging miR-330-5p. **(A)** The specific binding sites among HAGLROS, miR-330-5p and SPRR1B were predicted by the Lncbase database and the starBase database. **(B, C)** MiR-330-5p expression was examined in bladder tissues and cells using qRT–PCR assays. **(D)** The changes of miR-330-5p expression were determined using qRT–PCR assays. **(E)** FISH assay was used to confirm the subcellular colocalization between HAGLROS and miR-330-5p in BC cells (magnification, x 400). HAGLROS (Cy3, red), miR-330-5p (FAM, green), and cell nuclei (DAPI, blue). **(F)** FISH assay was used to confirm the subcellular colocalization between miR-330-5p and SPRR1B in BC cells (magnification, x 400). SPRR1B (Cy3, red), miR-330-5p (FAM, green), cell nuclei (DAPI, blue). **(G)** Wild-type or mutant HAGLROS was cotransfected with mimic-NC or miR-330-5p mimic into HEK-293T cells, and the relative luciferase activities were measured. **(H)** Wild-type or mutant SPRR1B was cotransfected with mimic-NC or miR-330-5p mimic into HEK-293T cells, and the relative luciferase activities were determined. Each experiment was repeated at least thrice. Ns, not significant. **P < 0.01, ***P < 0.001, ****P < 0.0001.

To study the regulatory mechanism of HAGLROS on SPRR1B, the potential miRNAs that shared putative binding sites with HAGLROS and SPRR1B cluster were predicted using the Lncbase database and the StarBase database. The data revealed that miR-330-5p shared putative binding sites with HAGLROS and SPRR1B cluster (**Figure 4A**). In addition, qRT–PCR assay was used to evaluate miR-330-5p expression levels in BC, and the results suggested that miR-330-5p was significantly downregulated in BC (**Figures 4B, C**). Furthermore, HAGLROS overexpression inhibited the expression of miR-330-5p, and HAGLROS knockdown increased the expression of miR-330-5p (**Figure 4D**). To further explore the relationship among HAGLROS, miR-330-5p and SPRR1B, FISH assays were performed to confirm their subcellular localizations in T24 and 5637 BC cells. The results indicate that all the above three genes are mainly located in the cytoplasm. In addition, HAGLROS may interact with miR-330-5p, and miR-330-5p could interact with SPRR1B (**Figures 4E, F**). Moreover, the dual-luciferase reporter assay data showed that HEK-293T cells cotransfected with wild-type HAGLROS and miR-330-5p mimics obviously decreased luciferase activity, but mut-type HAGLROS did not induce this change (**Figure 4G**). Additionally, HEK-293T cells cotransfected with wild-type SPRR1B and miR-330-5p mimics, but not mut-type SPRR1B, significantly inhibited the relative luciferase activity (**Figure 4H**). These results demonstrate that HAGLROS regulates the expression of SPRR1B by sponging miR-330-5p.

### Decreasing miR-330-5p Expression Reverses Malignant Phenotypes Inhabited by Silencing HAGLROS in BC Cells

**Figure 5:**
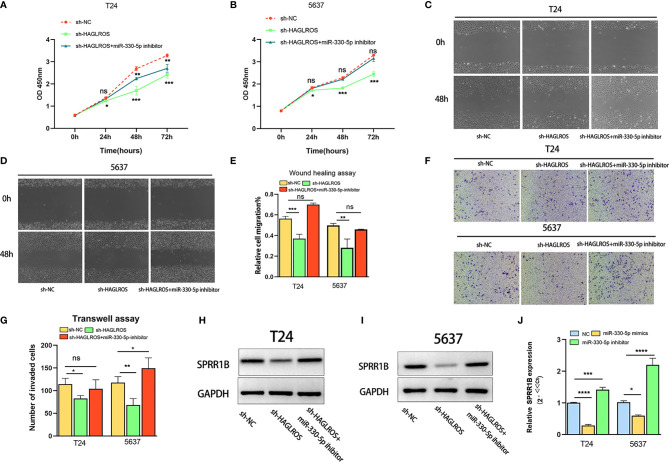
Decreased miR-330-5p reverses malignant phenotypes induced by silencing HAGLROS in BC cells. **(A, B)** The proliferation changes of T24 and 5637 BC cells were determined by CCK-8 assays. **(C–E)** The migration ability of T24 and 5637 BC cells were observed using wound healing assays (magnification, x40). **(F, G)** The invasion changes of T24 and 5637 BC cells were monitored using Transwell assays (magnification, x100). **(H, I)** The expression levels of SPRR1B were measured using western blot assays in T24 and 5637 BC cells. **(J)** The expression levels of SPRR1B were measured using qRT–PCR assays in T24 and 5637 BC cells. Each experiment was repeated at least three times. Ns, not significant. *P < 0.05, **P < 0.01, ***P < 0.001, ****P < 0.0001.

To further verify the regulatory mechanism of the HAGLROS/miR-330-5p/SPRR1B molecular axis, a series of gain- and loss-of-function assays were performed. The results showed that knockdown of miR-330-5p reversed the inhibitory effects on the proliferation, invasion and migration induced by silencing HAGLROS in BC cells (**Figures 5A–G**). In addition, the miR-330-5p inhibitor also reversed the expression of SPRR1B induced by silencing HAGLROS in BC cells (**Figures 5H, I**). Furthermore, the miR-330-5p mimic inhibited SPRR1B expression, whereas the miR-330-5p inhibitor promoted SPRR1B expression (**Figure 5J**). Notably, SPRR1B knockdown reversed the malignant phenotypes of BC cells promoted by decreasing miR-330-5p, thereby inhibiting the cancer-promoting effect of HAGLROS (**Supplementary Figure 3**). These results suggest that HAGLROS plays an important role in regulating the biological behaviour of BC by regulating the miR-330-5p/SPRR1B axis.

### HAGLROS Promotes the Growth of Bladder Cancer *In Vivo*

**Figure 6:**
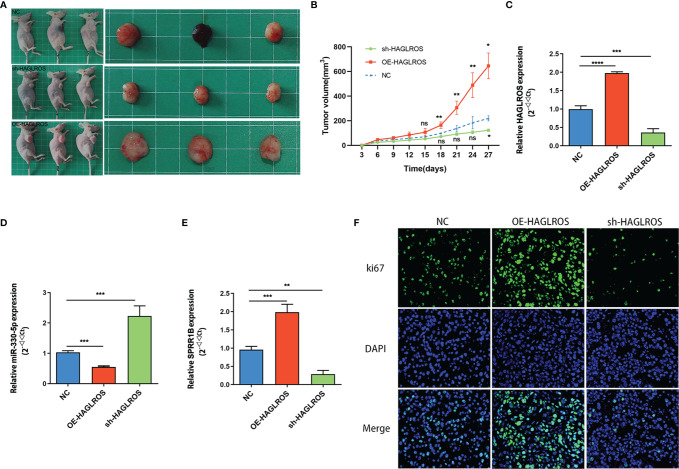
HAGLROS promotes the growth of BC *in vivo*. **(A)** T24 cells stably transfected with normal control, OE-HAGLROS and sh-HAGLROS vectors were used to establish subcutaneous xenograft mice model and the tumours collected from mouse were exhibited. **(B)** Tumour volumes were calculated every 3 days after injection. **(C–E)** The expression levels of HAGLROS, miR-330-5p and SPRR1B were measured by qRT-PCR assays. **(F)** The ki67 expression levels of the paraffin-embedded tumour tissues from nude mice were determined through immunofluorescence assay. Each experiment was repeated at least three times. Ns, not significant, *P < 0.05, **P < 0.01, ***P < 0.001, ****P < 0.0001.

To further validate whether HAGLROS regulates BC progression *in vivo*, T24 cells stably transfected with normal control, OE-HAGLROS and sh-HAGLROS vectors were used to establish subcutaneous xenograft mouse models. The results suggested that HAGLROS overexpression significantly promoted BC growth, while HAGLROS knockdown inhibited BC development compared with the control treatment (**Figures 6A, B**). The qRT-PCR results indicated that HAGLROS can also positively regulate SPRR1B expression and negatively regulate miR-330-5p expression *in vivo* (**Figures 6C–E**). Additionally, the expression level of ki67 was determined using an immunofluorescence assay. Consistently, the expression levels of ki-67 were dramatically increased in OE-HAGLROS group and obviously decreased in sh-HAGLROS group (**Figure 6F**). These data demonstrate HAGLROS can promote the growth of bladder tumour *in vivo*.

## Discussion

BC is the tenth most common malignant tumour and the fourteenth leading cause of cancer mortality worldwide and is characterized by a high recurrence rate, rapid progression and poor prognosis (1, 16). At present, the mechanisms involved in the pathogenesis and progression of BC remain unclear, and effective targets for early diagnosis and treatment of BC are urgently needed. In recent years, accumulating evidence has demonstrated that lncRNAs play an important regulatory role in cancers (6, 17, 18). Importantly, abnormal expression of lncRNAs is associated with increasing cancer-related mortality. For example, lncRNA CDKN2B-AS1 regulates progression and metastasis through the Cyclin-D pathway by interacting with miR-141 in renal cell carcinoma (19). LncRNA030, a novel lncRNA, maintains breast cancer stem cell stemness by stabilizing SQLE mRNA and increasing cholesterol synthesis (20). In addition, increasing studies have found that lncRNAs promote chemoresistance by regulating certain phase enzymes, altering drug efflux, repairing damaged DNA and inhibiting apoptosis in tumours (17). In the past decade, some of the significant lncRNAs were found to be related to BC incidence and development. For instance, UCA1 is specific and sensitive for the diagnosis of BC (21). Elevated H19 expression is associated with poor prognosis in BC (22).

HAGLROS, as a novel lncRNA, was first reported to promote the malignant phenotype of gastric cancer by sponging miR-100-5p to increase mTOR expression and interacting with mTORC1 components to activate the mTORC1 signalling pathway (13). Subsequently, numerous studies were performed to investigate the correlation between HAGLROS and cancers. For example, HAGLROS promotes proliferation, inhibits apoptosis and enhances autophagy by regulating the miR-5095/ATG12 axis and PI3K/AKT/mTOR signalling pathway in hepatocellular carcinoma (23). Lin et al. reported that knockdown HAGLROS can suppress the proliferation and promote the apoptosis of ovarian cancer cells by regulating miR-26b-5p, which affects the expression of apoptosis-related proteins (24). Notably, recent study indicated that HAGLROS was highly expressed and decreasing HAGLROS can inactivate the mTOR axis and elevate autophagy through improving lipid metabolism reprogramming in intrahepatic cholangiocarcinoma (25). In brief, growing evidence indicates that HAGLROS is closely related to apoptosis, autophagy, drug resistance and metabolic reprogramming and plays an oncogenic role in most cancers (26, 27). However, the biological functions and mechanisms of HAGLROS in BC remain unknown. In the present study, HAGLROS expression levels in BC were initially investigated using public databases, our transcriptome sequencing database, qRT–PCR and ISH assays. We found that HAGLROS is obviously increased in BC, and growing HAGLROS expression is correlated with poorer pathologic grade and clinical stage, which is significant for the diagnosis of BC. Moreover, a series of gain- and loss-of-function studies showed that HAGLROS can significantly promote proliferation, migration and invasion in BC.

The regulatory mechanism of lncRNAs depends in part on their subcellular localizations. Nuclear lncRNAs can regulate transcription by acting as enhancer RNAs (eRNAs), recruiting chromatin modifying complexes, regulating transcription factors, and cytoplasmic lncRNAs can regulate mRNA expression by regulating mRNA stability, mRNA translation, or competing microRNA binding (28). Our research shows that HAGLROS is mainly distributed in the cytoplasm of BC cells *via* subcellular fractionation assay and FISH assay. Therefore, we hypothesized that HAGLROS regulated BC growth and metastasis in a ceRNA-dependent manner, which is the most important and extensive regulatory mechanism of lncRNAs. Through comprehensive transcriptional analysis using TCGA dataset and our transcriptome dataset, we found that SPRR1B was significantly overexpressed, which was further confirmed in BC tissues and cells. In addition, our study also demonstrated that HAGLROS can positively regulate SPRR1B expression at both transcriptional and protein levels, and aberrantly expressed SPRR1B was related to malignant phenotypes in BC. These results suggest that SPRR1B is a downstream target protein of HAGLROS. To our knowledge, the present study is the first to initially explore the correlation between SPRR1B and bladder tumour. SPRR1B is a member of the SPRR family. Related studies have revealed that the SPRR1B is closely associated with the tumorigenesis and progression of carcinoma by regulating the epithelial-mesenchymal transition (EMT) and participating in the Ras/MEKK1/MKK1 and MAPK signalling pathways (29–33). These above research indicate the direction for further study of the mechanism of SPRR1B in BC.

To explore the regulatory mechanism of HAGLROS on SPRR1B, we used public databases to predict the potential miRNAs that shared putative binding sites with HAGLROS and SPRR1B cluster. We found that miR-330-5p shared putative binding sites with HAGLROS and SPRR1B cluster, which was confirmed through further experiments. Furthermore, our study revealed that miR-330-5p was decreased and could negatively regulate SPRR1B expression in BC. More importantly, we found that HAGLROS can negatively modulate the expression of miR-330-5p in BC. Rescue assays showed that decreased miR-330-5p reversed the inhibitory effects on cellular phenotypes induced by silencing HAGLROS in BC. Remarkably, silencing SPRR1B inhibited the regulatory functions of miR-330-5p knockdown on malignant phenotype in BC cells, thereby inhibiting the tumour-promoting effect of HAGLROS. These results provide strong evidence that HAGLROS can promote the malignant progression of BC by regulating the miR-330-5p/SPRR1B axis. Of note, numerous studies have shown that upregulated miR-330-5p can inhibit progression by interacting with proteins in a variety of tumours (34–37). For instance, Chen et al. found that miR-330-5p can suppress the progression of BC by inhibiting MTGR1 expression and the activity of the downstream Notch signalling pathway in BC (38). It is consistent with the results of our research. Our study also showed that miR-330-5p was significantly decreased in BC and could inhibit the proliferation, migration and invasion of BC cells by negatively regulating the expression of SPRR1B.

In summary, our study demonstrates that HAGLROS is significantly more highly expressed and plays an oncogenic role in BC. Mechanistically, we found that HAGLROS functions as a microRNA sponge to positively regulate SPRR1B expression by sponging miR-330-5p. Our findings help to elucidate the mechanism of the incidence and development of BC, and future studies will provide a powerful diagnostic and therapeutic target for BC. However, some limitations exist in the current research. For instance, we did not study the correlation between HAGLROS expression levels and BC patient prognosis due to the lack of sufficient quantities of long-term follow-up data. The regulatory mechanism of SPRR1B in BC should be investigated in further research.

## Data Availability Statement

The datasets presented in this article are not readily available because of relevant guidelines restrictions. Requests to access the datasets should be directed to the corresponding author/s.

## Ethics Statement

The studies involving human participants were reviewed and approved by the Medical Ethics Committee of the Second Affiliated Hospital of Kunming Medical University. The patients/participants provided their written informed consent to participate in this study.

## Author Contributions

Conceptualization ideas: JW, SX, and HW. Formal analysis: SX, SF, and DY. Investigation: SX, YL, and XQ. Methodology: SX, YZ, and YH. Project administration: JW and HW. Resources: JW and HW. Supervision: JW and HW. Verification: SX and HW. Writing - original draft: SX. Writing - review and editing: SX, JW, and HW. All authors contributed to the article and approved the submitted version.

## Funding

This work was supported by the National Natural Science Foundation of China (No. 82060464).

## Conflict of Interest

The authors declare that the research was conducted in the absence of any commercial or financial relationships that could be construed as a potential conflict of interest.

## Publisher’s Note

All claims expressed in this article are solely those of the authors and do not necessarily represent those of their affiliated organizations, or those of the publisher, the editors and the reviewers. Any product that may be evaluated in this article, or claim that may be made by its manufacturer, is not guaranteed or endorsed by the publisher.

## Supplementary Material

Supplementary Figure 1 Prediction of target mRNAs of HAGLROS. **(A)** The Venn diagram of upregulated DE-mRNAs in TCGA dataset and our RNA-sequencing dataset. **(B)** The top 10 upregulated DE-mRNAs in TCGA dataset and our RNA sequencing were intersected.

Supplementary Figure 2 Decreased SPRR1B inhibits HAGLROS expression in BC cells. **(A)** The expression levels of HAGLROS in T24 cells were measured by qRT-PCR assays. **(B)** The expression levels of HAGLROS in 5637 cells were detected through qRT-PCR assays. Ns, not significant, *****P*<0.0001.

Supplementary Figure 3 Knockdown of SPRR1B reverses the malignant phenotypes of BC cells promoted by decreasing miR-330-5p. **(A, B)** Cell proliferation was determined by CCK-8 assay in T24 and 5637 BC cells. **(C–E)** Cell migration was evaluated through wound healing assay in T24 and 5637 BC cells (magnification, x40). **(F–H)** Cell invasion was observed by transwell assays in T24 and 5637 BC cells (magnification, x100). Ns, not significant, **P*<0.05, ***P*<0.01, ****P*<0.001, *****P*<0.0001.

Supplementary Table 1 The sequence of lncRNA/mRNA primers.

Supplementary Table 2 Upregulated DE-lncRNAs in both TCGA dataset and RNA sequencing dataset.

Supplementary Table 3 Upregulated DE-mRNAs in both TCGA dataset and RNA sequencing dataset.
