# Supplementary figures and images for: Long Noncoding RNA HAGLROS Promotes the Malignant Progression of Bladder Cancer by Regulating the miR-330-5p/SPRR1B Axis
Source: Front Oncol. 2022 May 18;12:876090. doi: 10.3389/fonc.2022.876090 (PMC9159766; doi:10.3389/fonc.2022.876090)

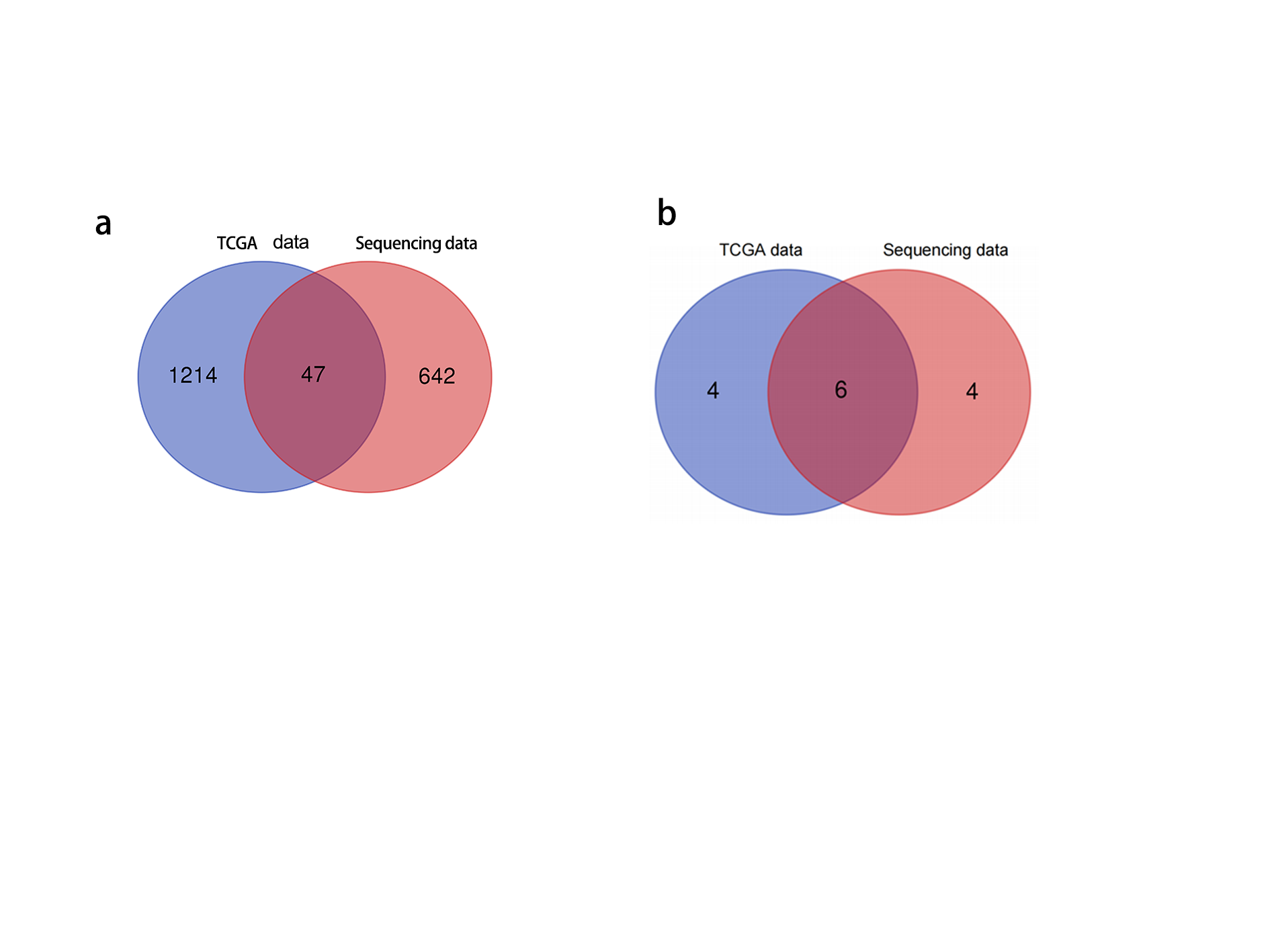

Supplement: Supplementary Figure 1 — Prediction of target mRNAs of HAGLROS. (A) The Venn diagram of upregulated DE-mRNAs in TCGA dataset and our RNA-sequencing dataset. (B) The top 10 upregulated DE-mRNAs in TCGA dataset and our RNA sequencing were intersected. [file Image_1.tif]

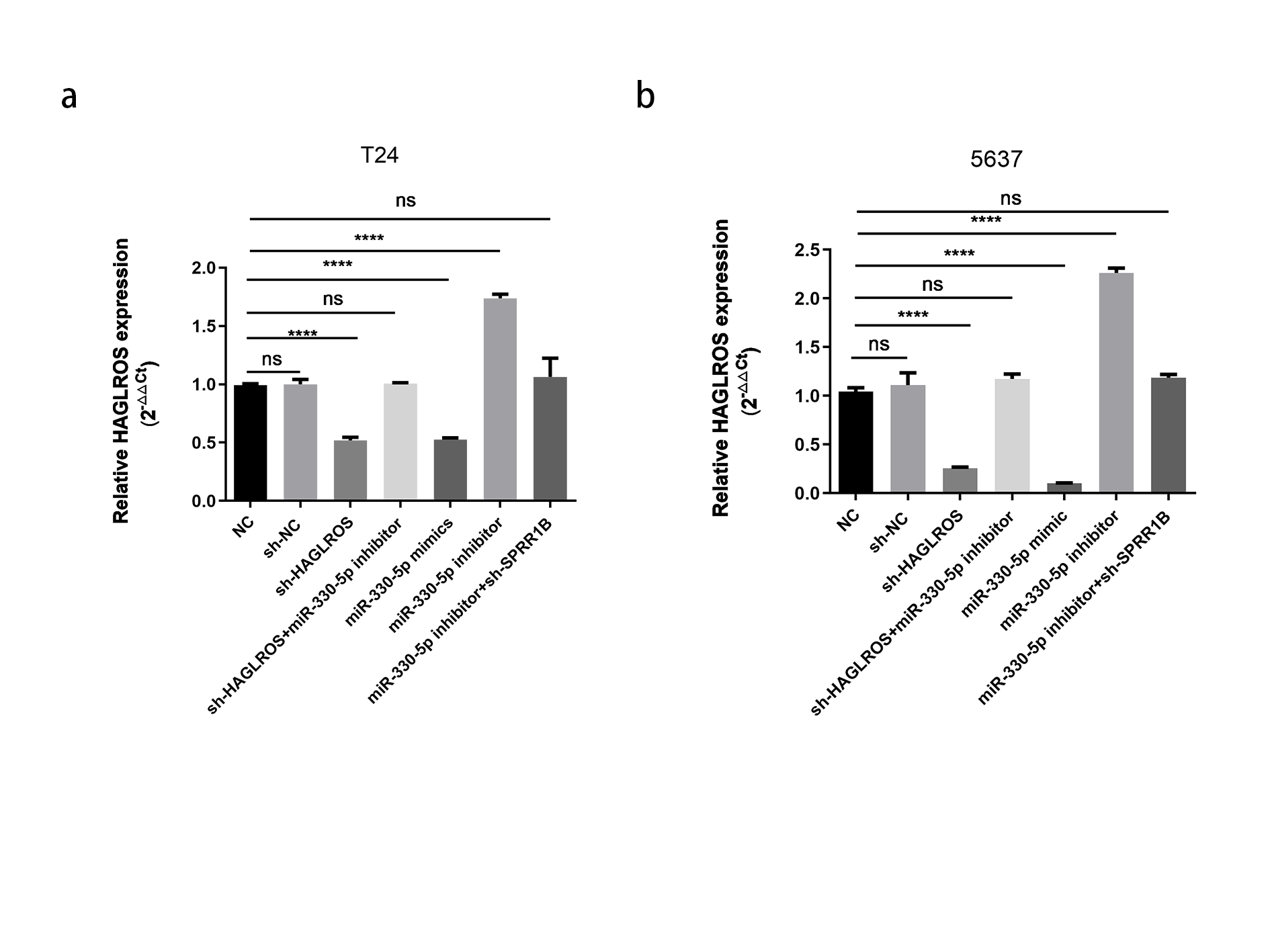

Supplement: Supplementary Figure 2 — Decreased SPRR1B inhibits HAGLROS expression in BC cells. (A) The expression levels of HAGLROS in T24 cells were measured by qRT-PCR assays. (B) The expression levels of HAGLROS in 5637 cells were detected through qRT-PCR assays. Ns, not significant, ****P<0.0001. [file Image_2.tif]

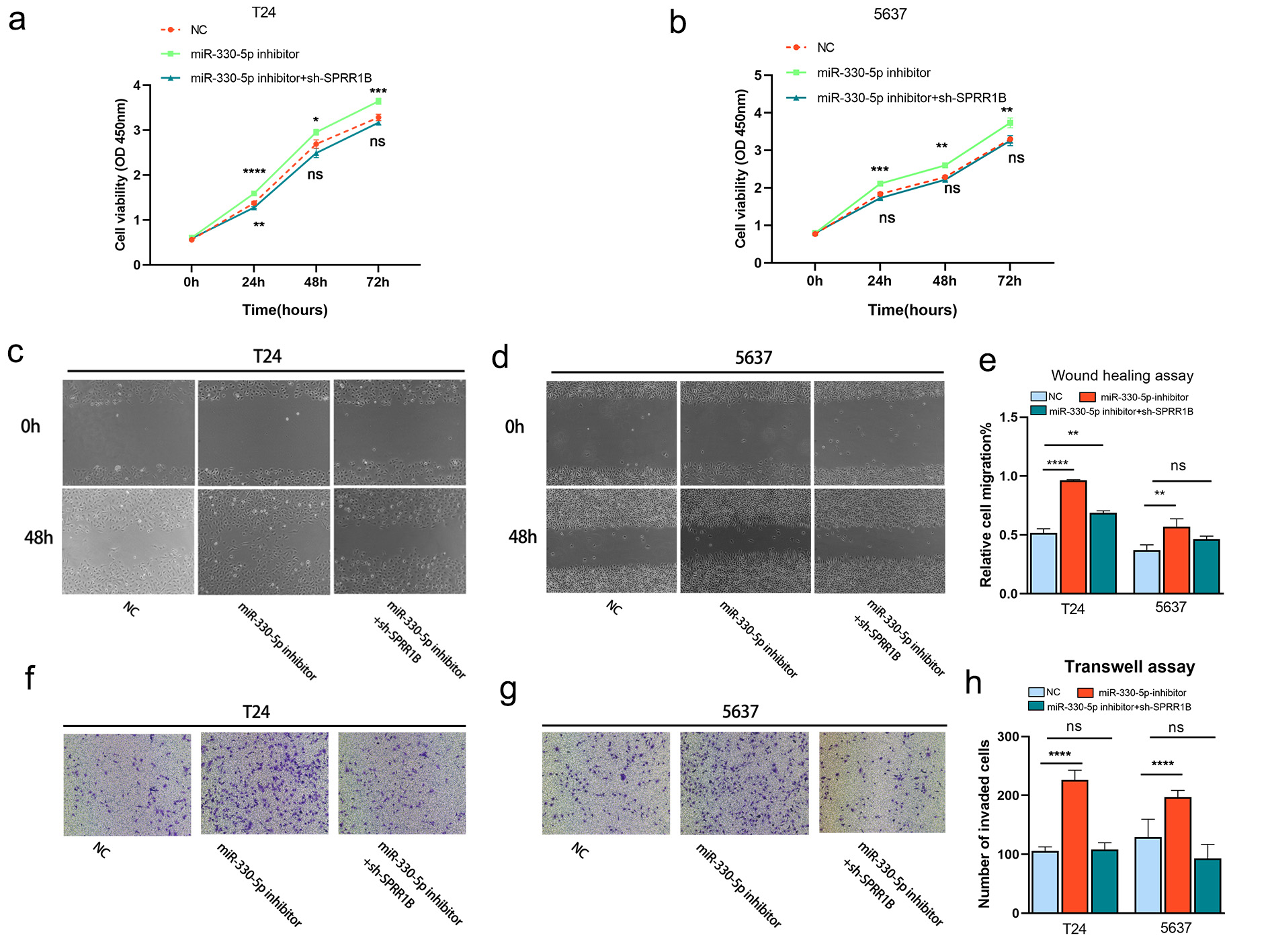

Supplement: Supplementary Figure 3 — Knockdown of SPRR1B reverses the malignant phenotypes of BC cells promoted by decreasing miR-330-5p. (A, B) Cell proliferation was determined by CCK-8 assay in T24 and 5637 BC cells. (C–E) Cell migration was evaluated through wound healing assay in T24 and 5637 BC cells (magnification, x40). (F–H) Cell invasion was observed by transwell assays in T24 and 5637 BC cells (magnification, x100). Ns, not significant, *P<0.05, **P<0.01, ***P<0.001, ****P<0.0001. [file Image_3.jpg]
