# Supplementary material for: Long Noncoding RNA HAGLROS Promotes the Malignant Progression of Bladder Cancer by Regulating the miR-330-5p/SPRR1B Axis
Source: Front Oncol. 2022 May 18;12:876090. doi: 10.3389/fonc.2022.876090 (PMC9159766; doi:10.3389/fonc.2022.876090)
Supplement: Supplementary Table 1 — The sequence of lncRNA/mRNA primers. [file Table_1.doc]

| **List of oligonucleotide sequences**  **Primers for PCR** | **5'--> 3'** |
| --- | --- |
| HAGLROS Forward | GACGAATACACCTCTGAA |
| HAGLROS Reverse | AGTCTTAGCCTACTTCCT |
| hsa-SPRR1B Forward | CAAGGTTCCAGAGCCATG |
| hsa-SPRR1B Reverse | TACTTCTGCTTGGTCTTCT |
| mmu-SPRR1B Forward | CAACCCATTGTTCCTGAG |
| mmu-SPRR1B Reverse | CTACTTTTGCTTTGTCTTCTG |
| hsa-GAPDH Forward | AAAGGGTCATCATCTCTG |
| hsa-GAPDH Reverse | GCTGTT GTCATACTTCTC |
| U6 Forward | CTCGCTTCGGCAGCACA |
| U6 Reverse | AACGCTTCACGAATTTGCGT |
| mmu-β-actin Forward | TATGGAATCCTGTGGCATC |
| mmu-β-actin Reverse | GTGTTGGCATAGAGGTCTT |
